# Impact of cigarette taxes on smoking prevalence from 2001-2015: A report using the Behavioral and Risk Factor Surveillance Survey (BRFSS)

**DOI:** 10.1371/journal.pone.0204416

**Published:** 2018-09-20

**Authors:** Michael S. Sharbaugh, Andrew D. Althouse, Floyd W. Thoma, Joon S. Lee, Vincent M. Figueredo, Suresh R. Mulukutla

**Affiliations:** 1 University of Pittsburgh Medical Center, Pittsburgh, Pennsylvania, United States of America; 2 Einstein Medical Center Philadelphia, Philadelphia, Pennsylvania, United States of America; University of Kentucky, UNITED STATES

## Abstract

**Objectives:**

To provide an up-to-date analysis on the relationship between excise taxes and the prevalence of cigarette smoking in the United States.

**Methods:**

Linear mixed-effects models were used to model the relationship between excise taxes and prevalence of cigarette smoking in each state from 2001 through 2015.

**Results:**

From 2001 through 2015, increases in state-level excise taxes were associated with declines in prevalence of cigarette smoking. The effect was strongest in young adults (age 18–24) and weakest in low-income individuals (<$25,000).

**Conclusions:**

Despite the shrinking pool of current smokers, excise taxes remain a valuable tool in public-health efforts to reduce the prevalence of cigarette smoking.

**Policy implications:**

States with high smoking prevalence may find increased excise taxes an effective measure to reduce population smoking prevalence. Since the effect is greatest in young adults, benefits of increased tax would likely accumulate over time by preventing new smokers in the pivotal young-adult years.

## Introduction

Tobacco smoking is associated with increased risk of a wide variety of health problems—certainly the best known being lung cancer and cardiovascular disease(s), but ongoing research now suggests that it affects nearly every organ in the body [1]. Many public health interventions have been attempted in efforts to decrease the population prevalence of cigarette smoking. Some examples include targeted smoking bans, advertising restrictions, health literacy campaigns, and excise taxes.

The smoking prevalence at any point in time reflects both the prior prevalence as well as the current rate of initiation and the current rate of cessation. While the prevalence of cigarette smoking in the United States (US) *has* decreased significantly in the past few decades [2], 15% of the adult population still identify as current smokers, according to a 2015 report from the Centers for Disease Control and Prevention (CDC) [3]. Furthermore, specific groups (i.e. males, aged 25–44 years, minority races, and lower socioeconomic status) were noted to have higher smoking prevalence.

There is conflicting evidence regarding the impact of pricing and/or taxes on smoking prevalence in the United States; some studies have suggested that increasing excise taxes are related to lower smoking prevalence, while others have suggested that taxation has minimal or no effect on smoking prevalence. Much research of the relationship between prices and smoking prevalence has been based on cross-sectional analyses, some using aggregate data [4, 5] while others used individual-level data [6–14]. In 1997, Meier and Licari performed a longitudinal analysis showing that increased excise taxes were related to declines in smoking in the United States from 1955–1994 [15]; we sought to build on this work with a more contemporary dataset.

The primary aim of this analysis was to assess the impact of changes in cigarette excise taxes on changes in smoking prevalence by state, utilizing publicly available data sources, from 2001–2015. As a secondary analysis, we also performed stratified analyses to determine whether the effects varied across age groups, race, and income level. Finally, we tested whether the impact of tax increases was influenced by prior tax rate or smoking prevalence.

## Methods

### Survey methodology

Smoking prevalence from 2001–2015 was estimated using data obtained from the Behavioral Risk Factor Surveillance System (BRFSS). The BRFSS is a telephone survey conducted annually by the CDC across all 50 states, the District of Columbia, and selected US territories. The BRFSS collects data regarding health-related behaviors, access to healthcare, and chronic conditions. A full description of the survey design, sampling methods, data collection, and statistical weighting can be found on the CDC website [16]. In 2011, the BRFSS methodology was expanded, beyond landlines, to include cellular phones while also updating the weighting methodology from poststratification weighting to iterative proportional fitting (raking) weighting [17].

From 2001–2004, current smokers were identified using the _SMOKER2 (Computed Smoking Status) variable, while from 2005–2015, the _SMOKER3 (Computed Smoking Status) variable was used. The responses of “Current Smoker—Now Smokes Every Day” and “Current Smoker—Now Smokes Some Days” were considered smokers in our analysis. The variable STOPSMK2 was used to identify if current smokers had tried to quit smoking in the prior 12 months.

Only respondents with a reported age were included in the analysis. Age classifications were identified using the _AGEG5YR (Reported age in five-year age categories) variable from the 2001–2015 BRFSS data. From there, Age was further classified into four age categories; 18–24, 25–44, 45–65, and 65+ years of age.

Reported household income was identified in the 2001–2015 datasets using the INCOME2 (Computed income categories) variable. Household income was grouped into four categories; <$25,000, $25,000-<$50,000, $50,000-<$75,000, and ≥$75,000.

Races included for sub-population analysis were White/Non-Hispanic, Black/Non-Hispanic, and Hispanic. Race was identified from 2001–2012 with the _RACEGR2 (Five level race/ethnicity category) variable and from 2013–2015 with the _RACEGR3 (Five level race/ethnicity category) variable.

### Cigarette taxes

State excise taxes (per pack of cigarettes) from 2001–2015 were collected from the Federation of Tax Administrators [18]. This analysis included only taxes imposed at the state level, but does not account for any additional county or city taxes. Any further “in lieu” cigarette sales tax or surcharges were also excluded.

### Statistical methods

All statistical analyses were performed using SAS version 9.4 (SAS Institute, Cary, NC). The SURVEYFREQ procedure was utilized to apply appropriate weight, strata, and cluster variables to calculate the prevalence of current smokers and the percentage of current smokers who tried to quit within the past 12 months by state for each year from 2001–2015. Linear mixed-effects models were used to estimate the absolute percent change in smoking prevalence associated with an increase of $0.25 in cigarette tax to correspond with an amount that most people can easily relate. Smoking prevalence in each state was the dependent variable, with a fixed-effect for the state’s per-pack cigarette tax and random effects for state and year. Analyses were performed using the entire BRFSS cohort and then stratified by age, gender, income, and race to determine whether the effect of cigarette taxes on smoking prevalence varied across different sociodemographic groups. The models are presented such that the reader can see the estimated mean change in overall smoking prevalence associated with a 25-cent tax increase, then see the effects of the same 25-cent tax increase on smoking prevalence within specific groups of interest (age, gender, income, and race). In each model, interaction terms (tax increase*prior smoking, tax increase*prior tax rate) were tested to determine whether the relationship between tax increases and smoking prevalence was affected by prior tax rate or prior smoking prevalence.

## Results

### Trends in smoking prevalence, 2001–2015

**Fig 1:**
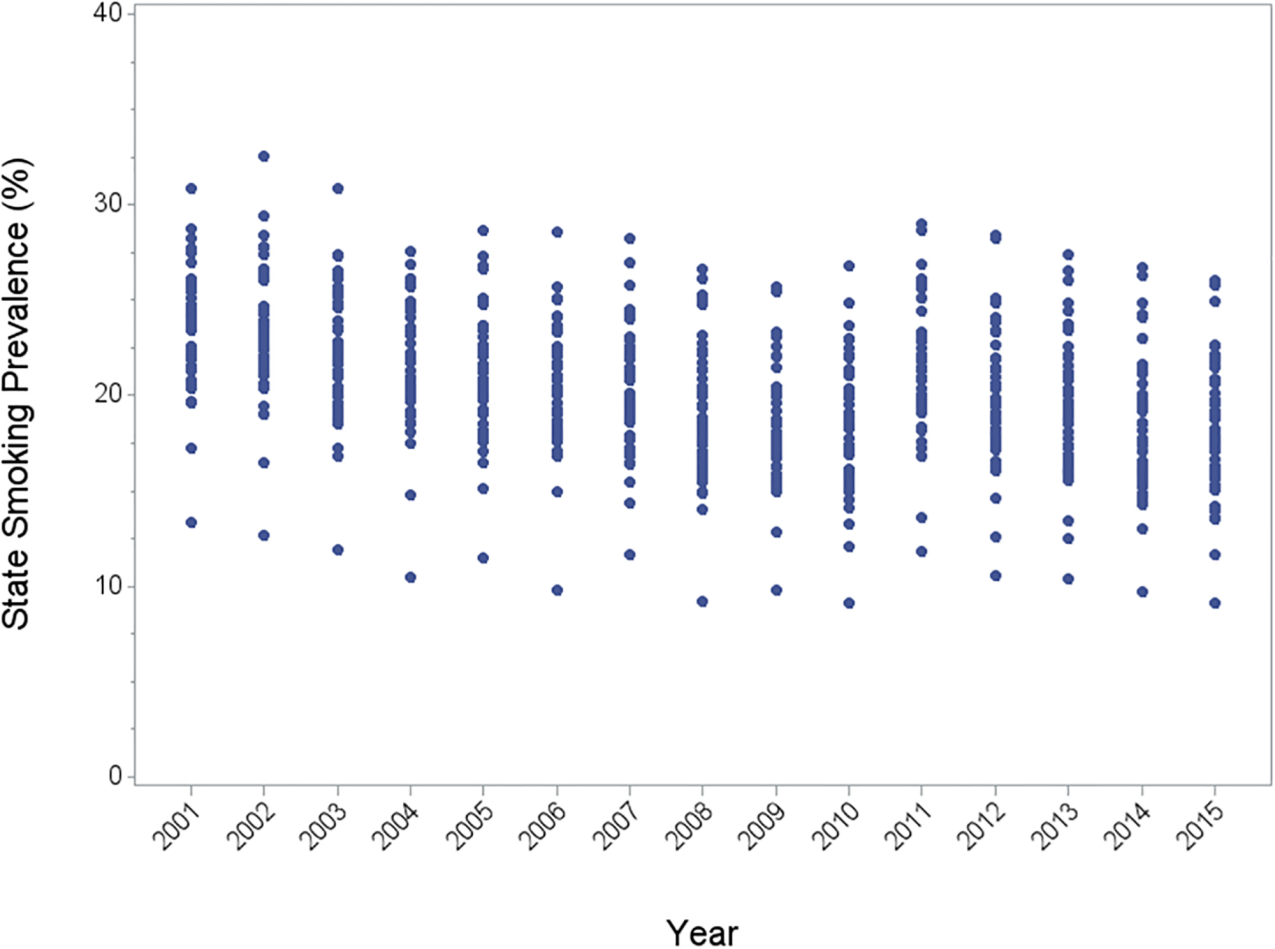
Distribution of state level smoking prevalence, 2001–2015.

Over 5.5 million adults participated in the BRFSS from 2001–2015 (S1 Table). State level smoking prevalence ranged from 13.3–30.9% in 2001; this decreased to range of 9.1–26.1% in 2015 (Fig 1; for details see S2 Table). During the same period, there was an increase in current smokers who tried to quit within the prior 12 months; in 2001 the state level rates of current smokers that reported trying to quit ranged from 47.7–65.7%, while in 2015 the range was 53.3–68.3%.

### Trends in cigarette taxes, 2001–2015

**Fig 2:**
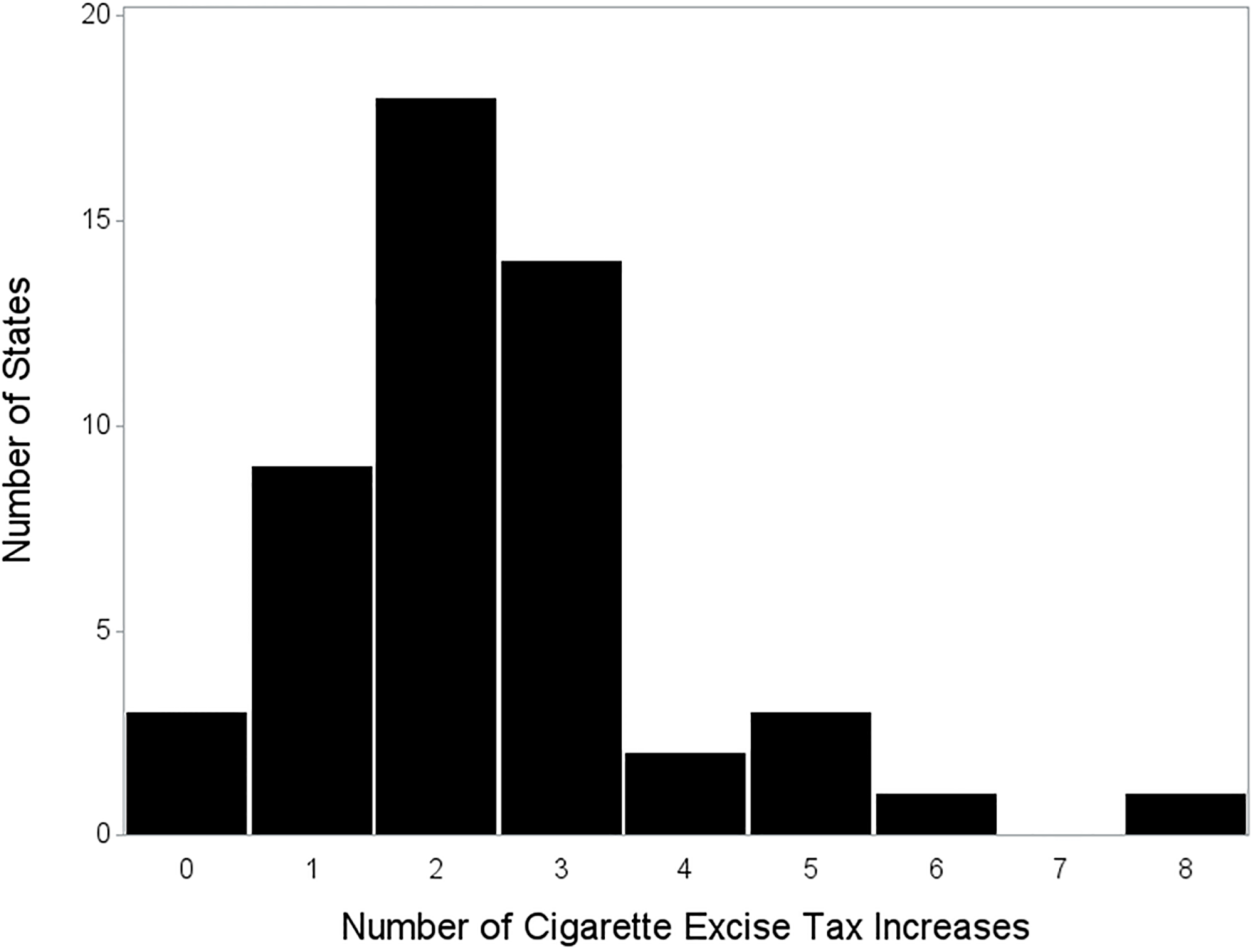
Frequency of increases in state level cigarette tax, 2001–2015.

**Fig 3:**
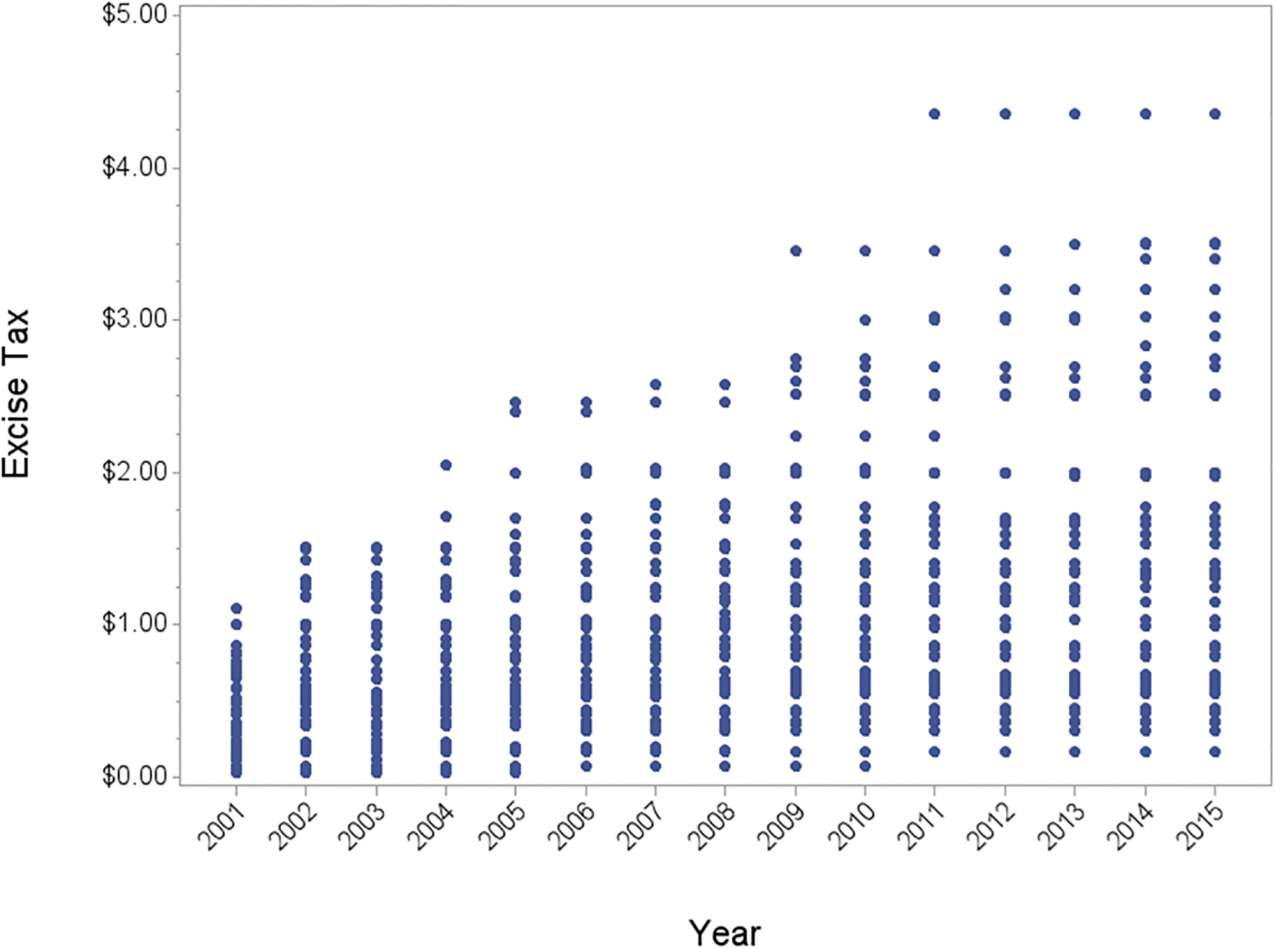
Distribution of state level cigarette taxes, 2001–2015.

The frequency of cigarette tax increases by state is summarized in Fig 2. Three states did not increase cigarette taxes at all from 2001–2015; most states increased cigarette taxes either twice (18 states) or three times (14 states); and five states increased cigarette taxes 5 times or more. In 2001, state cigarette taxes ranged from $0.03-$1.11 per pack; by 2015, the range had increased to $0.17-$4.35 per pack (Fig 3; for details see S3 Table).

### Impact of cigarette tax increases on smoking prevalence

Linear mixed-effects models suggest that an additional $0.25 per-pack tax was associated with an estimated 0.6% absolute reduction in smoking prevalence (p<0.001; Table 1). Notably, increases in cigarette tax had the greatest impact on smoking prevalence in younger adults; a $0.25 per-pack tax increase was associated with an estimated 1.5% reduction in smoking prevalence among those aged 18–24 (p<0.001), 0.5% reduction in ages 25–44; 0.4% reduction in ages 45–64; and 0.2% reduction in those 65+ years of age, respectively.

**Table 1:** Model-based estimated impact of $0.25 tax increase on smoking prevalence.

	Smoking Prevalence			Tried to Quit Smoking within the Past 12 Months		
	Beta	95% CI	p-value	Beta	95% CI	p-value
All Participants	-0.60	(-0.66, -0.55)	<0.001	0.67	(0.57, 0.76)	<0.001
Stratified By Age						
18–24	-1.45	(-1.59, -1.30)	<0.001	0.29	(0.09, 0.48)	0.003
25–44	-0.50	(-0.58, -0.42)	<0.001	0.85	(0.72, 0.97)	<0.001
45–64	-0.41	(-0.47, -0.35)	<0.001	0.87	(0.74, 1.00)	<0.001
65+	-0.19	(-0.23, -0.16)	<0.001	0.53	(0.35, 0.71)	<0.001
Stratified By Gender						
Male	-0.58	(-0.66, -0.51)	<0.001	0.68	(0.55, 0.80)	<0.001
Female	-0.60	(-0.66, -0.54)	<0.001	0.63	(0.51, 0.73)	<0.001
Stratified By Income						
<$25,000	-0.09	(-0.17, -0.01)	0.030	0.70	(0.57, 0.83)	<0.001
$25,000-<$50,000	-0.75	(-0.82, -0.67)	<0.001	0.66	(0.52, 0.79)	<0.001
$50,000-<$75,000	-0.63	(-0.71, -0.56)	<0.001	0.63	(0.47, 0.79)	<0.001
≥$75,000	-0.62	(-0.68, -0.55)	<0.001	0.65	(0.48, 0.82)	<0.001
Stratified By Race/Ethniciy						
White Only, Non-Hispanic	-0.63	(-0.69, -0.57)	<0.001	0.60	(0.51, 0.70)	<0.001
Black Only, Non-Hispanic	-0.41	(-0.64, -0.19)	0.0002	0.20	(-0.13, 0.52)	0.237
Hispanic	-0.65	(-0.83, -0.48)	<0.001	0.58	(0.28, 0.87)	0.0001

Paradoxically, when stratifying by income, cigarette taxes had the least impact in smoking prevalence for those with the lowest income, with a $0.25 tax increase associated with a minimal change (0.09% reduction) in smoking prevalence among participants with income <$25,000; in contrast, there was an estimated 0.75% reduction per $0.25 tax increase in those making $25,000-$50,000; a 0.63% reduction per $0.25 tax increase in those making $50,000-$75,000; and a 0.62% reduction per $0.25 tax increase in those making ≥$75,000.

When stratifying by race, the difference between groups was noticeable but somewhat less pronounced. Estimated responses to a $0.25 tax increase were very similar in White/Non-Hispanic (0.63% reduction in smoking prevalence) and Hispanic (0.65% reduction) subgroups while the Black/Non-Hispanic subgroup had an estimated 0.4% reduction in response to $0.25 per-pack tax increase.

The results were virtually identical for males and females. There appeared to be no differential effect of a tax increase on smoking prevalence by gender.

We also tested for interactions between tax increase and two potential effect modifiers thought to affect the possible relationship between tax increases and smoking prevalence: each state’s prior smoking prevalence and prior tax rate. Interestingly, the interaction term between tax increase and prior smoking prevalence had a significant (p = 0.001) positive association with smoking prevalence, suggesting that the effect of tax increases was more pronounced in states with a higher baseline smoking prevalence. However, there was no significant interaction between the effect of a tax increase and the prior tax rate (p = 0.298), indicating that the effects of an excise tax increase on smoking prevalence were not dependent on the prior tax rate in a given state and year.

### Impact of cigarette taxes on attempts to quit

It appears that increased taxes also have an association with the percentage of smokers that tried to quit the past 12 months. Our analyses suggest that an additional $0.25 in cigarette tax was associated with an estimated 0.67% increase in the percentage of active smokers that reported trying to quit smoking (Table 1). This effect was most pronounced in participants aged 25–44 (0.85% increase per $0.25 additional tax) and 45–64 (0.87% increase per $0.25 additional tax). The impact of an additional $0.25 tax is relatively similar across income levels, with the smallest effect being 0.63% for those making $50,000-<$75,000, while the largest absolute increase is 0.70% for those making <$25,000. Finally, when stratifying by race, we again see that White/Non-Hispanic (0.60%) and Hispanic (0.58%) have similar absolute increases in the percentage of smokers who tried to quit, while Black/Non-Hispanic has the smallest estimated increase of 0.2% in response to the additional tax.

## Discussion

In this manuscript, we have used data from a nationally representative survey, the Behavior Risk Factor Surveillance System, to evaluate the relationship between changes in state cigarette taxes and smoking prevalence. Our analysis of a contemporary dataset of the US population has five main points. First, there is an inverse relationship between cigarette taxes and smoking prevalence; specifically, that each additional $0.25 per-pack in excise tax is associated with a 0.6% reduction in smoking prevalence. Second, an increase in cigarette excise taxes appeared to have the greatest effect on smoking prevalence in younger adults aged 18–24, with an estimated 1.5% reduction in smoking prevalence per $0.25 additional tax in this age group. Third, increases in cigarette taxes appear to have the greatest effect on smoking prevalence in higher-income populations, while the effect was much more muted in the lowest-income population. Fourth, increases in excise taxes also appear to increase the reported prevalence of efforts to quit smoking, suggesting that the decline in smoking prevalence may be a combination of discouraging new smokers from starting as well as encouraging current smokers to quit or attempt to quit. Fifth, the effect of a state-level tax increase was consistent regardless of the state’s prior tax rate.

One particularly interesting finding is that excise taxes had the greatest effect on smoking prevalence in the 18–24 age group, consistent with prior findings [6]. This is especially encouraging considering data from the National Survey on Drug Use and Health (2012), which suggested that 98% of adult smokers in the United States started smoking before age 26, and that very little initiation of cigarette smoking begins in adulthood.^1^ Taken together, one can argue that reducing smoking prevalence in the young-adult population, when most smokers initiate their smoking habit, is the most effective target group to achieve and sustain reductions in population smoking prevalence. Furthermore, if cigarette taxes have the greatest immediate influence on smoking prevalence in the young adult population, it is possible that the full effects of cigarette taxes on population smoking prevalence cannot by fully appreciated until several years after the tax is implemented, and that prior cross-sectional studies of the relationship between cigarette taxes and smoking prevalence have underestimated the impact of excise taxes on long-term smoking prevalence.

Another informative finding is that cigarette taxes seemed to have the least impact on smoking prevalence in lower-income participants, with just a 0.09% estimated decrease in smoking with each $0.25 increase in excise tax for the lowest-income group (<$25,000) in comparison to 0.6–0.7% decrease per $0.25 increase for the three higher income subgroups. Although possibly confounded by education and other unmeasured factors, this finding suggests that interventions other than excise taxes may be needed to meaningfully reduce the smoking prevalence in lower-income populations. Recent data from the Minnesota Adult Tobacco Survey found that socially disadvantaged smokers were most likely to respond to tax increases by adopting price-minimization behaviors (i.e. rolling their own cigarettes, buying cheaper brands, or buying from cheaper places) without sustained cessation, which is one potential explanation for the apparently modest effect of tax increases on smoking prevalence in the low-SES population [19, 20]. It’s also likely that low-income individuals who attempt to quit may not have access to therapeutic options to help them with smoking cessation, such as nicotine-replacement products (patch, gum, lozenges, inhalers, or nasal sprays).

Interestingly, in our analyses, the effect of excise taxes on the proportion of smokers that reported *attempting to quit* was consistent across all income strata, so perhaps the resistance to tax in the low-income population is a combination of i) lack of sustained cessation in the low-SES population that attempts to quit and ii) a greater percentage of new-onset smokers (rather than just a lack of smoking cessation efforts) in this population. Therefore, public-health efforts in low-SES areas should i) provide resources to low-SES smokers that express interest in attempting cessation and ii) target young adults to prevent the onset of new smoking in the most vulnerable and impactful population.

While the magnitude of the relationship varied across different populations, it is encouraging to note that excise taxes were associated with decreased smoking prevalence and with increased attempts to quit across all subgroups examined. Although some individual states may have seen decreases in smoking prevalence independent of any tax increase, our modeling suggests that when pooling data across the 50 states, those with greater tax increases tended to experience the largest decline in smoking prevalence. Prior studies, conducted in varied populations, have suggested that excise taxes and other public health interventions are correlated with lower population smoking prevalence [5–14], and thus our broad-spectrum findings of a negative association between taxes and smoking prevalence are unsurprising. However, given that most prior studies were performed using cross-sectional analyses, it is interesting to see this confirmed by analyses of serial ecological data using a contemporary dataset from the United States. We also acknowledge that the analyses performed here do not account for changes in federal taxes as well as other locally-implemented sales taxes, nor do we account for the implementation of smoking-reduction efforts in specific states other than taxes; there may be other factors associated with decrease in smoking prevalence that we could not account for in the analyses, such as changes in smoke-free laws [21]. A prior CDC report shows that the number of states with comprehensive smoke-free laws (worksites, restaurants, and bars) increased from zero on December 31, 2000 to 26 states on December 31, 2010 while many other states introduced partial bans or restrictions. It is possible that concomitant introduction of smoke-free laws in states that were also increasing taxes may have had an effect above and beyond that of tax increases on smoking prevalence. Finally, we elected not to include federal taxes as these would have been uniform across the 50 states. However, our data suggest that given the lack of relationship between the absolute taxation rate and the effect of tax increase, individual states have the opportunity to use state-level policy to affect smoking prevalence, even against the backdrop of any federal policy. Finally, we were unable to model more granular changes in smoking behavior (i.e. total daily consumption or modifications in buying patterns) as the BRFSS ceased recording the total number of cigarettes smoked per day in 2000.

## Conclusion

Our contemporary analysis based on 15 years of recent data suggests that a $0.25 increase in state excise tax is associated with a 0.6% decrease in population smoking prevalence, and that the effects are especially pronounced in young adults. Cigarette excise taxes should remain a key element of public health policy in the ongoing efforts to eradicate tobacco smoking and thus improve population health.

## Supporting information

S1 Table Number of BRFSS participants 2001–2015. (DOCX)

S2 Table Cigarette smoking prevalence by state, 2001–2015. (DOCX)

S3 Table Per-pack cigarette taxes by state, 2001–2015. (DOCX)

## References

[1] U.S. Department of Health and Human Services. The Health Consequences of Smoking: 50 Years of Progress. A Report of the Surgeon General. Atlanta, GA: U.S. Department of Health and Human Services, Centers for Disease Control and Prevention, National Center for Chronic Disease Prevention and Health Promotion, Office on Smoking and Health, 2014.

[2] Dwyer-Lindgren L, Mokdad AH, Srebotnjak T. Flaxman AD, Hansen GM, Murray CJL. Cigarette smoking prevalence in US counties: 1996–2012. *Population Health Metrics* 2014; 12: 5 10.1186/1478-7954-12-5 24661401 PMC3987818

[3] Jamal A, King BA, Neff LJ, Whitmill J, Babb SD, Graffunder CM. Current Cigarette Smoking Among Adults—United States, 2005–2015. *MMWR* 2016; 65: 1205–1211. doi: 10.15585/mmwr.mm6544a2 27832052 10.15585/mmwr.mm6544a2

[4] Baltagi BH, Levin D. Estimating dynamic demand for cigarettes using panel data: the effects of bootlegging, taxation and advertising reconsidered. *Rev Econ Stat* 1986; 68 (1); 148–155.

[5] Chapman S, Richardson J. Tobacco excise and declining consumption: the case of Papua New Guinea. *Am J Public Health* 1990; 80; 537–540 2327528 10.2105/ajph.80.5.537 PMC1404661

[6] Lewit EM, Coate D. The potential for using excise taxes to reduce smoking. *J Health Econ* 1982; 1, 121–145 10263952 10.1016/0167-6296(82)90011-x

[7] Wasserman J, Manning WG, Newhouse JP, Winkler JD. The effects of excise taxes and regulations on cigarette smoking. *J Health Econ* 1991; 10(1), 43–64 10112149 10.1016/0167-6296(91)90016-g

[8] Chaloupka FJ. Rational addictive behavior and cigarette smoking. *J Polit Econ* 1991; 99, 722–742

[9] Chaloupka FJ, Wechsler H. Price, tobacco control policies and smoking among young adults. *J Health Econ* 1997; 16; 359–373. 10169306 10.1016/s0167-6296(96)00530-9

[10] Decicca P, Kenkel D, Mathios A. Putting out the fires: will higher taxes reduce the onset of youth smoking? *J Polit Econ* 2002; 110(1); 144–169.

[11] Carpenter C, Cook P. Cigarette taxes and youth smoking; new evidence from national, state, and local youth risk behavior surveys. *J Health Econ* 2008; 27; 287–299. 10.1016/j.jhealeco.2007.05.008 18242745

[12] Decicca P, Kenkel D, Mathios A, Shin Y, Lim J. Youth smoking, cigarette prices, and anti-smoking sentiment. *Health Econ* 2008; 17(6); 733–749. 10.1002/hec.1293 17935201

[13] Kostova D, Ross H, Blecher E, Markowitz S. Is youth smoking responsive to cigarette prices? Evidence from low- and middle-income countries. *Tob Control* 2011; 20; 419–424. 10.1136/tc.2010.038786 21737858

[14] Vuolo M, Kelly BC, Kadowaki J. Independent and Interactive Effects of Smoking Bans and Tobacco Taxes on a Cohort of US Young Adults. *Am J Pub Health* 2016; 106(2); 374–380. 26691133 10.2105/AJPH.2015.302968 PMC4758814

[15] Meier KJ, Licari MJ. The Effect of Cigarette Taxes on Cigarette Consumption, 1955 through 1994. *Am J Pub Health* 1997; 87(7): 1126–1130. 9240101 10.2105/ajph.87.7.1126 PMC1380885

[16] http://www.cdc.gov/brfss/.

[17] Centers for Disease Control and Prevention. Methodologic changes in the behavioral risk factor surveillance system in 2011 and potential effects on prevalence estimates. *MMWR* 2012; 61: 410–413. 22672976

[18] https://www.taxadmin.org/current-tax-rates.

[19] Choi K, Boyle RG. Changes in cigarette expenditure minimizing strategies before and after a cigarette tax increase. *Tob Control* 2017 (epub ahead of print) 10.1136/tobaccocontrol-2016-053415 PMC5585063 28219975

[20] Parks MJ, Kingsbury JH, Boyle RG, Choi K. Behavioral change in response to a statewide tobacco tax increase and differences across socioeconomic status. *Addict Behav* 2017; 73: 209–215. 10.1016/j.addbeh.2017.05.019 28551589 PMC5510536

[21] Tynan M, Babb S, MaNeil A, Griffin M. State Smoke-Free Laws for Worksites, Restaurants, and Bars—United States, 2000–2010. *MMWR* 2011; 60(15): 472–475. 21508923

