# Supplementary material for: Impact of cigarette taxes on smoking prevalence from 2001-2015: A report using the Behavioral and Risk Factor Surveillance Survey (BRFSS)
Source: PLoS One. 2018 Sep 20;13(9):e0204416. doi: 10.1371/journal.pone.0204416 (PMC6147505; doi:10.1371/journal.pone.0204416)
Supplement: S1 Table — (DOCX) [file pone.0204416.s001.docx]

**S1 Table.** Number of BRFSS Participants 2001-2015

| Year | Unweighted | Weighted |
| --- | --- | --- |
| 2001 | 203,021 | 210,015,179 |
| 2002 | 238,852 | 212,200,393 |
| 2003 | 255,657 | 215,997,876 |
| 2004 | 295,027 | 217,313,301 |
| 2005 | 347,278 | 219,950,795 |
| 2006 | 344,487 | 222,800,879 |
| 2007 | 420,217 | 225,790,935 |
| 2008 | 403,191 | 228,478,872 |
| 2009 | 420,968 | 230,653,439 |
| 2010 | 440,788 | 232,566,209 |
| 2011 | 493,064 | 233,448,803 |
| 2012 | 462,810 | 238,455,434 |
| 2013 | 479,201 | 241,266,775 |
| 2014 | 450,432 | 243,018,259 |
| 2015 | 429,089 | 245,920,119 |
