# Supplementary material for: Impact of cigarette taxes on smoking prevalence from 2001-2015: A report using the Behavioral and Risk Factor Surveillance Survey (BRFSS)
Source: PLoS One. 2018 Sep 20;13(9):e0204416. doi: 10.1371/journal.pone.0204416 (PMC6147505; doi:10.1371/journal.pone.0204416)
Supplement: S2 Table — (DOCX) [file pone.0204416.s002.docx]

**S2 Table.** Cigarette Smoking Prevalence By State, 2001-2015

| State | 2001 | 2002 | 2003 | 2004 | 2005 | 2006 | 2007 | 2008 | 2009 | 2010 | 2011 | 2012 | 2013 | 2014 | 2015 |
| --- | --- | --- | --- | --- | --- | --- | --- | --- | --- | --- | --- | --- | --- | --- | --- |
| Alaska | 23.9% | 24.4% | 25.3% | 24.9% | 24.8% | 23.3% | 22.5% | 22.2% | 22.6% | 22.0% | 24.4% | 23.8% | 21.6% | 21.2% | 21.4% |
| Alabama | 26.1% | 29.4% | 26.3% | 24.9% | 25.0% | 24.2% | 22.2% | 21.7% | 20.5% | 20.4% | 22.9% | 20.6% | 22.6% | 20.1% | 19.2% |
| Arkansas | 21.5% | 23.5% | 21.0% | 18.6% | 20.4% | 18.1% | 19.8% | 15.9% | 16.2% | 15.0% | 19.2% | 17.2% | 16.4% | 16.5% | 14.2% |
| Arizona | 25.6% | 26.3% | 24.8% | 25.7% | 23.5% | 23.7% | 22.4% | 22.4% | 21.5% | 23.0% | 26.9% | 25.1% | 26.1% | 24.8% | 24.9% |
| California | 17.2% | 16.4% | 16.8% | 14.8% | 15.2% | 14.9% | 14.3% | 14.0% | 12.9% | 12.1% | 13.6% | 12.6% | 12.5% | 13.0% | 11.7% |
| Colorado | 22.4% | 20.4% | 18.5% | 20.1% | 19.9% | 17.9% | 18.7% | 17.6% | 17.1% | 16.0% | 18.3% | 17.8% | 17.8% | 15.8% | 15.8% |
| Connecticut | 20.8% | 19.5% | 18.7% | 18.1% | 16.5% | 17.0% | 15.5% | 16.0% | 15.5% | 13.3% | 17.2% | 16.1% | 15.6% | 15.5% | 13.6% |
| District of Columbia | 25.1% | 24.7% | 21.9% | 24.5% | 20.7% | 21.7% | 19.0% | 17.8% | 18.4% | 17.3% | 21.8% | 19.8% | 19.7% | 20.0% | 17.5% |
| Delaware | 20.8% | 20.4% | 22.3% | 21.0% | 20.1% | 17.9% | 17.3% | 16.4% | 15.3% | 15.7% | 20.8% | 19.8% | 18.8% | 16.5% | 16.2% |
| Florida | 22.4% | 22.1% | 23.9% | 20.4% | 21.6% | 21.0% | 19.3% | 17.5% | 17.2% | 17.2% | 19.4% | 17.6% | 16.9% | 17.8% | 15.8% |
| Georgia | 23.7% | 23.3% | 22.8% | 20.1% | 22.2% | 20.0% | 19.3% | 19.5% | 17.7% | 17.7% | 21.3% | 20.4% | 18.9% | 17.4% | 17.8% |
| Hawaii | 20.6% | 21.1% | 17.3% | 0.0% | 17.1% | 17.5% | 17.0% | 15.4% | 15.3% | 14.5% | 16.8% | 14.6% | 13.4% | 14.3% | 14.1% |
| Iowa | 19.7% | 20.6% | 19.0% | 17.5% | 17.9% | 16.8% | 19.2% | 16.9% | 16.3% | 15.7% | 17.2% | 16.3% | 17.3% | 16.0% | 13.9% |
| Idaho | 23.6% | 22.8% | 23.4% | 22.2% | 19.9% | 20.5% | 20.2% | 21.3% | 18.6% | 16.9% | 20.9% | 18.6% | 18.1% | 16.6% | 15.1% |
| Illinois | 27.5% | 27.7% | 26.1% | 25.0% | 27.3% | 24.1% | 24.1% | 26.1% | 23.2% | 21.2% | 25.7% | 24.1% | 22.0% | 23.0% | 20.7% |
| Indiana | 22.2% | 23.1% | 21.7% | 20.8% | 20.4% | 21.5% | 19.8% | 18.8% | 17.2% | 16.1% | 20.3% | 18.2% | 19.5% | 18.5% | 18.1% |
| Kansas | 22.2% | 22.1% | 20.4% | 19.8% | 17.8% | 20.0% | 17.9% | 17.9% | 17.8% | 17.0% | 22.0% | 19.5% | 20.1% | 18.2% | 17.8% |
| Kentucky | 30.9% | 32.6% | 30.8% | 27.6% | 28.7% | 28.6% | 28.3% | 25.3% | 25.7% | 24.8% | 29.0% | 28.4% | 26.6% | 26.3% | 26.1% |
| Louisiana | 24.8% | 23.9% | 26.6% | 23.6% | 22.6% | 23.4% | 22.6% | 20.5% | 22.1% | 22.2% | 25.7% | 24.9% | 23.6% | 24.1% | 21.9% |
| Massachusetts | 24.0% | 23.6% | 23.6% | 21.0% | 20.9% | 20.9% | 20.1% | 18.2% | 17.3% | 18.2% | 22.8% | 20.3% | 20.3% | 19.3% | 19.5% |
| Maryland | 21.3% | 22.0% | 20.2% | 19.7% | 19.0% | 17.8% | 17.1% | 14.9% | 15.3% | 15.3% | 19.2% | 16.2% | 16.5% | 14.7% | 15.2% |
| Maine | 19.6% | 19.0% | 19.2% | 18.5% | 18.1% | 17.8% | 16.4% | 16.1% | 15.0% | 14.2% | 18.3% | 16.5% | 16.7% | 14.9% | 14.1% |
| Michigan | 25.7% | 24.2% | 26.2% | 23.4% | 22.1% | 22.4% | 21.2% | 20.4% | 19.6% | 19.0% | 23.3% | 23.4% | 21.5% | 21.3% | 20.8% |
| Minnesota | 22.2% | 21.7% | 21.1% | 20.7% | 20.0% | 18.3% | 16.5% | 17.6% | 16.8% | 15.0% | 19.1% | 18.8% | 18.1% | 16.3% | 16.3% |
| Missouri | 25.4% | 27.4% | 25.6% | 24.6% | 23.7% | 25.1% | 24.0% | 22.7% | 23.4% | 23.0% | 26.0% | 24.0% | 24.9% | 23.0% | 22.6% |
| Mississippi | 25.9% | 26.6% | 27.3% | 24.1% | 23.4% | 23.3% | 24.6% | 25.0% | 23.1% | 21.1% | 25.1% | 24.0% | 22.2% | 20.6% | 22.5% |
| Montana | 21.9% | 21.3% | 19.9% | 20.4% | 19.2% | 19.0% | 19.5% | 18.5% | 16.9% | 18.8% | 22.1% | 19.7% | 19.0% | 20.0% | 19.0% |
| North Carolina | 20.3% | 22.8% | 21.3% | 20.3% | 21.3% | 18.6% | 19.9% | 18.4% | 16.7% | 17.2% | 20.0% | 19.7% | 18.5% | 17.4% | 17.1% |
| North Dakota | 26.9% | 26.0% | 25.2% | 23.2% | 23.1% | 22.2% | 21.5% | 22.3% | 22.1% | 21.4% | 23.0% | 18.3% | 19.5% | 17.1% | 17.7% |
| Nebraska | 24.1% | 23.2% | 21.2% | 21.8% | 20.5% | 18.7% | 19.4% | 17.0% | 15.8% | 16.9% | 19.5% | 17.3% | 16.3% | 17.6% | 16.0% |
| New Hampshire | 21.3% | 19.1% | 19.5% | 18.9% | 18.1% | 18.1% | 17.2% | 14.8% | 15.9% | 14.5% | 16.9% | 17.4% | 15.7% | 15.2% | 13.6% |
| New Jersey | 23.9% | 21.2% | 22.0% | 20.3% | 21.5% | 20.2% | 20.8% | 19.4% | 17.9% | 18.5% | 21.5% | 19.4% | 19.2% | 19.2% | 17.6% |
| New Mexico | 23.4% | 22.4% | 21.6% | 20.0% | 20.5% | 18.3% | 18.9% | 16.8% | 18.1% | 15.5% | 18.2% | 16.3% | 16.7% | 14.5% | 15.3% |
| Nevada | 25.9% | 26.4% | 24.8% | 23.2% | 22.7% | 22.1% | 22.9% | 20.9% | 20.4% | 19.9% | 21.8% | 20.9% | 20.3% | 19.2% | 19.1% |
| New York | 22.1% | 21.5% | 20.5% | 19.9% | 20.0% | 19.6% | 21.0% | 18.2% | 18.6% | 17.4% | 21.9% | 21.3% | 21.2% | 19.8% | 18.8% |
| Ohio | 27.7% | 26.6% | 25.4% | 25.9% | 22.3% | 22.5% | 23.1% | 20.2% | 20.4% | 22.5% | 25.1% | 23.4% | 23.4% | 21.1% | 21.7% |
| Oklahoma | 28.8% | 26.7% | 25.2% | 26.1% | 25.1% | 25.1% | 25.8% | 24.8% | 25.5% | 23.7% | 26.1% | 23.3% | 23.7% | 21.2% | 22.1% |
| Oregon | 20.5% | 22.4% | 21.0% | 20.0% | 18.5% | 18.5% | 16.9% | 16.3% | 17.9% | 15.1% | 19.8% | 17.9% | 17.4% | 17.1% | 17.2% |
| Pennsylvania | 24.6% | 24.6% | 25.5% | 22.7% | 23.7% | 21.5% | 20.9% | 21.4% | 20.2% | 18.5% | 22.5% | 21.4% | 21.1% | 20.0% | 18.2% |
| Rhode Island | 24.0% | 22.5% | 22.4% | 21.3% | 19.8% | 19.3% | 17.0% | 17.4% | 15.2% | 15.7% | 20.1% | 17.6% | 17.4% | 16.3% | 15.6% |
| South Carolina | 26.2% | 26.6% | 25.5% | 24.5% | 22.6% | 22.3% | 21.9% | 20.1% | 20.3% | 21.0% | 23.2% | 22.6% | 22.0% | 21.6% | 19.8% |
| South Dakota | 22.4% | 22.6% | 22.7% | 20.3% | 19.8% | 20.4% | 19.8% | 17.6% | 17.5% | 15.4% | 23.1% | 22.0% | 19.7% | 18.6% | 20.2% |
| Tennessee | 24.4% | 27.8% | 25.7% | 26.1% | 26.8% | 22.6% | 24.3% | 23.2% | 22.1% | 20.1% | 23.1% | 25.0% | 24.4% | 24.2% | 21.9% |
| Texas | 22.5% | 22.9% | 22.1% | 20.6% | 20.0% | 18.1% | 19.4% | 18.6% | 18.0% | 15.9% | 19.2% | 18.2% | 16.0% | 14.7% | 15.3% |
| Utah | 13.3% | 12.7% | 12.0% | 10.5% | 11.5% | 9.8% | 11.7% | 9.2% | 9.8% | 9.2% | 11.8% | 10.6% | 10.4% | 9.7% | 9.1% |
| Virginia | 22.4% | 21.2% | 19.6% | 20.0% | 19.3% | 18.0% | 17.6% | 16.8% | 17.2% | 15.4% | 19.1% | 16.5% | 16.6% | 16.4% | 16.1% |
| Vermont | 22.5% | 24.6% | 22.1% | 20.9% | 20.6% | 19.3% | 18.6% | 16.5% | 19.2% | 18.6% | 20.9% | 19.1% | 19.1% | 19.6% | 16.7% |
| Washington | 22.6% | 21.5% | 19.5% | 19.2% | 17.6% | 17.1% | 16.8% | 15.7% | 15.0% | 15.2% | 17.6% | 17.3% | 16.1% | 15.3% | 15.1% |
| Wisconsin | 28.2% | 28.4% | 27.4% | 26.9% | 26.6% | 25.7% | 27.0% | 26.6% | 25.6% | 26.8% | 28.6% | 28.3% | 27.4% | 26.7% | 25.8% |
| West Virginia | 23.6% | 23.3% | 22.1% | 22.0% | 20.8% | 20.8% | 19.6% | 19.9% | 18.8% | 19.1% | 20.9% | 20.4% | 18.8% | 17.4% | 17.3% |
| Wyoming | 22.2% | 23.7% | 24.6% | 21.7% | 21.3% | 21.6% | 22.1% | 19.4% | 19.9% | 19.5% | 23.1% | 21.9% | 20.8% | 19.7% | 19.1% |
