# Supplementary material for: Impact of cigarette taxes on smoking prevalence from 2001-2015: A report using the Behavioral and Risk Factor Surveillance Survey (BRFSS)
Source: PLoS One. 2018 Sep 20;13(9):e0204416. doi: 10.1371/journal.pone.0204416 (PMC6147505; doi:10.1371/journal.pone.0204416)
Supplement: S3 Table — (DOCX) [file pone.0204416.s003.docx]

**S3 Table.** Per-Pack Cigarette Taxes By State, 2001-2015

| State | 2001 | 2002 | 2003 | 2004 | 2005 | 2006 | 2007 | 2008 | 2009 | 2010 | 2011 | 2012 | 2013 | 2014 | 2015 |
| --- | --- | --- | --- | --- | --- | --- | --- | --- | --- | --- | --- | --- | --- | --- | --- |
| Alaska | $1.00 | $1.00 | $1.00 | $1.00 | $1.60 | $1.60 | $1.80 | $2.00 | $2.00 | $2.00 | $2.00 | $2.00 | $2.00 | $2.00 | $2.00 |
| Alabama | $0.17 | $0.17 | $0.17 | $0.17 | $0.43 | $0.43 | $0.43 | $0.43 | $0.43 | $0.43 | $0.43 | $0.43 | $0.43 | $0.43 | $0.43 |
| Arkansas | $0.32 | $0.59 | $0.34 | $0.59 | $0.59 | $0.59 | $0.59 | $0.59 | $1.15 | $1.15 | $1.15 | $1.15 | $1.15 | $1.15 | $1.15 |
| Arizona | $0.58 | $1.18 | $1.18 | $1.18 | $1.18 | $1.18 | $2.00 | $2.00 | $2.00 | $2.00 | $2.00 | $2.00 | $2.00 | $2.00 | $2.00 |
| California | $0.87 | $0.87 | $0.87 | $0.87 | $0.87 | $0.87 | $0.87 | $0.87 | $0.87 | $0.87 | $0.87 | $0.87 | $0.87 | $0.87 | $0.87 |
| Colorado | $0.20 | $0.20 | $0.20 | $0.20 | $0.87 | $0.84 | $0.84 | $0.84 | $0.84 | $0.84 | $0.84 | $0.84 | $0.84 | $0.84 | $0.84 |
| Connecticut | $0.50 | $1.51 | $1.11 | $1.51 | $1.51 | $1.51 | $1.51 | $2.00 | $2.00 | $3.00 | $3.00 | $3.00 | $3.00 | $3.40 | $3.40 |
| District of Columbia | $0.65 | $1.00 | $1.00 | $1.00 | $1.00 | $1.00 | $1.00 | $1.00 | $2.00 | $2.50 | $2.50 | $2.50 | $2.50 | $2.50 | $2.50 |
| Delaware | $0.24 | $0.55 | $0.24 | $0.55 | $0.55 | $0.55 | $0.55 | $1.15 | $1.15 | $1.60 | $1.60 | $1.60 | $1.60 | $1.60 | $1.60 |
| Florida | $0.34 | $0.34 | $0.34 | $0.34 | $0.34 | $0.34 | $0.34 | $0.34 | $1.34 | $1.34 | $1.34 | $1.34 | $1.34 | $1.34 | $1.34 |
| Georgia | $0.12 | $0.37 | $0.12 | $0.37 | $0.37 | $0.37 | $0.37 | $0.37 | $0.37 | $0.37 | $0.37 | $0.37 | $0.37 | $0.37 | $0.37 |
| Hawaii | $1.00 | $1.30 | $1.20 | $1.30 | $1.40 | $1.40 | $1.60 | $1.80 | $2.60 | $2.60 | $3.00 | $3.20 | $3.20 | $3.20 | $3.20 |
| Iowa | $0.36 | $0.36 | $0.36 | $0.36 | $0.36 | $0.36 | $0.36 | $1.36 | $1.36 | $1.36 | $1.36 | $1.36 | $1.36 | $1.36 | $1.36 |
| Idaho | $0.28 | $0.57 | $0.28 | $0.57 | $0.57 | $0.57 | $0.57 | $0.57 | $0.57 | $0.57 | $0.57 | $0.57 | $0.57 | $0.57 | $0.57 |
| Illinois | $0.58 | $0.98 | $0.98 | $0.98 | $0.98 | $0.98 | $0.98 | $0.98 | $0.98 | $0.98 | $0.98 | $0.98 | $1.98 | $1.98 | $1.98 |
| Indiana | $0.16 | $0.56 | $0.56 | $0.56 | $0.56 | $0.56 | $0.56 | $1.00 | $1.00 | $1.00 | $1.00 | $1.00 | $1.98 | $1.00 | $1.00 |
| Kansas | $0.24 | $0.79 | $0.70 | $0.79 | $0.79 | $0.79 | $0.79 | $0.79 | $0.79 | $0.79 | $0.79 | $0.79 | $0.79 | $0.79 | $0.79 |
| Kentucky | $0.03 | $0.03 | $0.03 | $0.03 | $0.03 | $0.30 | $0.30 | $0.30 | $0.60 | $0.60 | $0.60 | $0.60 | $0.60 | $0.60 | $0.60 |
| Louisiana | $0.24 | $0.36 | $0.36 | $0.36 | $0.36 | $0.36 | $0.36 | $0.36 | $0.36 | $0.36 | $0.36 | $0.36 | $0.36 | $0.36 | $0.36 |
| Massachusetts | $0.76 | $1.51 | $1.51 | $1.51 | $1.51 | $1.51 | $1.51 | $1.51 | $2.51 | $2.51 | $2.51 | $2.51 | $2.51 | $3.51 | $3.51 |
| Maryland | $0.66 | $1.00 | $1.00 | $1.00 | $1.00 | $1.00 | $1.00 | $2.00 | $2.00 | $2.00 | $2.00 | $2.00 | $2.00 | $2.00 | $2.00 |
| Maine | $0.74 | $1.00 | $1.00 | $1.00 | $1.00 | $2.00 | $2.00 | $2.00 | $2.00 | $2.00 | $2.00 | $2.00 | $2.00 | $2.00 | $2.00 |
| Michigan | $0.75 | $1.25 | $1.25 | $1.25 | $2.00 | $2.00 | $2.00 | $2.00 | $2.00 | $2.00 | $2.00 | $2.00 | $2.00 | $2.00 | $2.00 |
| Minnesota | $0.48 | $0.48 | $0.48 | $0.48 | $0.48 | $1.23 | $1.23 | $1.23 | $1.23 | $1.23 | $1.23 | $1.23 | $1.23 | $2.83 | $2.90 |
| Missouri | $0.17 | $0.17 | $0.17 | $0.17 | $0.17 | $0.17 | $0.17 | $0.17 | $0.17 | $0.17 | $0.17 | $0.17 | $0.17 | $0.17 | $0.17 |
| Mississippi | $0.18 | $0.18 | $0.18 | $0.18 | $0.18 | $0.18 | $0.18 | $0.18 | $0.68 | $0.68 | $0.68 | $0.68 | $0.68 | $0.68 | $0.68 |
| Montana | $0.18 | $0.70 | $0.18 | $0.70 | $1.70 | $1.70 | $1.70 | $1.70 | $1.70 | $1.70 | $1.70 | $1.70 | $1.70 | $1.70 | $1.70 |
| North Carolina | $0.05 | $0.05 | $0.05 | $0.05 | $0.05 | $0.30 | $0.35 | $0.35 | $0.35 | $0.45 | $0.45 | $0.45 | $0.45 | $0.45 | $0.45 |
| North Dakota | $0.44 | $0.44 | $0.44 | $0.44 | $0.44 | $0.44 | $0.44 | $0.44 | $0.44 | $0.44 | $0.44 | $0.44 | $0.44 | $0.44 | $0.44 |
| Nebraska | $0.34 | $0.64 | $0.64 | $0.64 | $0.64 | $0.64 | $0.64 | $0.64 | $0.64 | $0.64 | $0.64 | $0.64 | $0.64 | $0.64 | $0.64 |
| New Hampshire | $0.52 | $0.52 | $0.52 | $0.52 | $0.52 | $0.80 | $0.80 | $1.08 | $1.78 | $1.78 | $1.78 | $1.68 | $1.68 | $1.78 | $1.78 |
| New Jersey | $0.80 | $1.50 | $1.50 | $2.05 | $2.40 | $2.40 | $2.58 | $2.58 | $2.70 | $2.70 | $2.70 | $2.70 | $2.70 | $2.70 | $2.70 |
| New Mexico | $0.21 | $0.91 | $0.21 | $0.91 | $0.91 | $0.91 | $0.91 | $0.91 | $0.91 | $0.91 | $1.66 | $1.66 | $1.66 | $1.66 | $1.66 |
| Nevada | $0.65 | $0.35 | $0.35 | $0.80 | $0.80 | $0.80 | $0.80 | $0.80 | $0.80 | $0.80 | $0.80 | $0.80 | $0.80 | $0.80 | $0.80 |
| New York | $1.11 | $1.50 | $1.50 | $1.50 | $1.50 | $1.50 | $1.50 | $1.50 | $2.75 | $2.75 | $4.35 | $4.35 | $4.35 | $4.35 | $4.35 |
| Ohio | $0.23 | $0.55 | $0.55 | $0.55 | $0.55 | $1.25 | $1.25 | $1.25 | $1.25 | $1.25 | $1.25 | $1.25 | $1.25 | $1.25 | $1.25 |
| Oklahoma | $0.23 | $0.23 | $0.23 | $0.23 | $1.03 | $1.03 | $1.03 | $1.03 | $1.03 | $1.03 | $1.03 | $1.03 | $1.03 | $1.03 | $1.03 |
| Oregon | $0.68 | $1.28 | $1.28 | $1.28 | $1.18 | $1.18 | $1.18 | $1.18 | $1.18 | $1.18 | $1.18 | $1.18 | $1.18 | $1.31 | $1.31 |
| Pennsylvania | $0.31 | $1.00 | $1.00 | $1.00 | $1.35 | $1.35 | $1.35 | $1.35 | $1.35 | $1.60 | $1.60 | $1.60 | $1.60 | $1.60 | $1.60 |
| Rhode Island | $0.71 | $1.50 | $1.32 | $1.71 | $2.46 | $2.46 | $2.46 | $2.46 | $3.46 | $3.46 | $3.46 | $3.46 | $3.50 | $3.50 | $3.50 |
| South Carolina | $0.07 | $0.07 | $0.07 | $0.07 | $0.07 | $0.07 | $0.07 | $0.07 | $0.07 | $0.07 | $0.57 | $0.57 | $0.57 | $0.57 | $0.57 |
| South Dakota | $0.33 | $0.53 | $0.33 | $0.53 | $0.53 | $0.53 | $0.53 | $1.53 | $1.53 | $1.53 | $1.53 | $1.53 | $1.53 | $1.53 | $1.53 |
| Tennessee | $0.13 | $0.20 | $0.20 | $0.20 | $0.20 | $0.20 | $0.20 | $0.62 | $0.62 | $0.62 | $0.62 | $0.62 | $0.62 | $0.62 | $0.62 |
| Texas | $0.41 | $0.41 | $0.41 | $0.41 | $0.41 | $0.41 | $1.41 | $1.41 | $1.41 | $1.41 | $1.41 | $1.41 | $1.41 | $1.41 | $1.41 |
| Utah | $0.52 | $0.70 | $0.70 | $0.70 | $0.70 | $0.70 | $0.70 | $0.70 | $0.70 | $0.70 | $1.70 | $1.70 | $1.70 | $1.70 | $1.70 |
| Virginia | $0.03 | $0.03 | $0.03 | $0.03 | $0.20 | $0.30 | $0.30 | $0.30 | $0.30 | $0.30 | $0.30 | $0.30 | $0.30 | $0.30 | $0.30 |
| Vermont | $0.44 | $1.19 | $0.93 | $1.19 | $1.19 | $1.19 | $1.79 | $1.79 | $2.24 | $2.24 | $2.24 | $2.62 | $2.62 | $2.62 | $2.75 |
| Washington | $0.83 | $1.43 | $1.43 | $1.43 | $1.43 | $2.03 | $2.03 | $2.03 | $2.03 | $2.03 | $3.03 | $3.03 | $3.03 | $3.03 | $3.03 |
| Wisconsin | $0.59 | $0.77 | $0.77 | $0.77 | $0.77 | $0.77 | $0.77 | $1.77 | $2.52 | $2.52 | $2.52 | $2.52 | $2.52 | $2.52 | $2.52 |
| West Virginia | $0.17 | $0.55 | $0.17 | $0.55 | $0.55 | $0.55 | $0.55 | $0.55 | $0.55 | $0.55 | $0.55 | $0.55 | $0.55 | $0.55 | $0.55 |
| Wyoming | $0.12 | $0.60 | $0.12 | $0.60 | $0.60 | $0.60 | $0.60 | $0.60 | $0.60 | $0.60 | $0.60 | $0.60 | $0.60 | $0.60 | $0.60 |
